# Mukbang and Disordered Eating: A Netnographic Analysis of Online Eating Broadcasts

**DOI:** 10.1007/s11013-020-09674-6

**Published:** 2020-04-10

**Authors:** Mattias Strand, Sanna Aila Gustafsson

**Affiliations:** 1 Stockholm Centre for Eating Disorders, Wollmar Yxkullsgatan 27B, 118 50 Stockholm, Sweden; 2 Centre for Psychiatry Research, Department of Clinical Neuroscience, Karolinska Institutet, & Stockholm Health Care Services, Stockholm County Council, 171 77 Stockholm, Sweden; 3 University Health Care Research Center, Faculty of Medicine and Health, Örebro University, 701 82 Örebro, Sweden

**Keywords:** Eating disorders, Disordered eating, Binge eating, Body image, Social media

## Abstract

Mukbang is a recent Internet phenomenon in which video recordings of hosts eating large amounts of food are streamed on an online video platform. It originated in South Korea around 2014 and has since become a global trend. The aim of this study was to explore how viewers of mukbang videos relate their audience experiences to symptoms of disordered eating. A qualitative analysis of YouTube comments and Reddit posts on the topic of mukbang and disordered eating was performed, employing a netnographic approach. Two overarching themes were identified: a viewer perspective, by which users discuss mukbang without describing any personal involvement, and a participant perspective, by which users describe their own experiences of affects and behaviors in response to watching mukbang. Several topical categories emerged, describing how watching mukbang can both limit and increase eating, reduce loneliness and guilt, and become self-destructive. For some, mukbang appears to be a constructive tool in increasing food intake, preventing binge eating, or reducing loneliness; for others, it is clearly a destructive force that may motivate restrictive eating or trigger a relapse into loss-of-control eating. Notably, watching mukbang is not necessarily experienced as either helpful or destructive, but instead as simultaneously useful and hurtful.

## Introduction: South Korean Eating Broadcasts Gone Global

The word *mukbang* (먹방; also stylized as *muk-bang* or *meokbang*) is a Korean abbreviation for ‘eating show’—a concise summary of this recent Internet phenomenon. A mukbang is a video recording of a host occasionally preparing or arranging but most importantly eating large amounts of food, usually while leisurely chatting with the audience, that is streamed or posted on an online video platform. The amounts of food ingested in a typical mukbang are conspicuously large; sometimes this involves large amounts of a single food-item (such as ramen noodles or fried chicken) but a mukbang may, for example, also consist of multiple double-portion dishes.

Many South Korean mukbang hosts originally started their broadcasting careers on the Korean live video platform AfreecaTV. However, the mukbang phenomenon has rapidly spread on a global level and a large number of mukbang videos can also be found on internationally oriented platforms such as YouTube, where they are not necessarily live-streamed. According to Google Trends, mukbang started trending as an online search term in South Korea during Winter 2014 and globally in Autumn 2014 (Google Trends 2018).

In some cases, Korean mukbang hosts on AfreecaTV earn money for their ‘performance’ by viewer contributions in virtual currency; not seldom, hosts can be heard naming and thanking viewers in real time when they notice that someone has contributed money. Reportedly, the most popular mukbang hosts can earn as much as 10,000 United States dollars a month (Quartz 2016; Chu 2018). It can be assumed that some of this income finances further food purchases, such as in mukbangs involving large amounts of sushi or shellfish. However, most foods consumed in mukbang videos appear to be relatively cheap; e.g., multiple packs of instant ramen noodles. Notably, South Korea has in the past years had the world’s fastest wireless Internet service and WI-FI hubs are ubiquitous, which has contributed to transforming Korean video streaming from desktop-based to mobile-based and to the further integration of online media into most aspects of life (Quartz 2016). In this context, mukbang has also been described as the most recent tool in promoting Korean cuisine abroad and as a vital part of *Hallyu*, or the Korean Wave, a term referring to the phenomenal international growth of Korean popular culture encompassing, for example, K-pop music, art house cinema, and television dramas since the 1990s and onwards (Chu 2018).

Mukbang has been understood as originating in “the loneliness of unmarried or uncoupled Koreans, in addition to the inherently social aspects of eating in Korea” (Quartz 2016). In Korean society, dining is a deeply social activity and many Koreans find it almost unthinkable to sit and eat alone, not least in public; hence, mukbang has been described as a virtual substitute for socializing while eating on your own (Jackson 2018). In parallel with this tendency, South Korean health officials have expressed great concern with a recent surge in obesity among Koreans and have suggested that phenomena such as mukbang could require more attention and possibly even some form of government regulation (Chu 2018).

The mukbang phenomenon shares some common characteristics with the so-called *cheatmeals*—i.e., large meals that people that follow a strict diet and/or an intensive physical exercise regime occasionally ‘allow’ themselves as a reward or an incentive—that have previously been described and analyzed in relation to disordered eating (Pila et al. 2017). Furthermore, many mukbang videos are perceived and labeled by users as ‘ASMR’, which is short for *autonomous sensory meridian response*: a sought-after tingling sensation in the crown of the head upon experiencing or watching certain intricate audio-visual triggers such as gentle whispering, the turning of book pages, or somebody slurping and chewing in a delicate manner (Poerio et al. 2018).

Disordered eating is a broad term describing various types of problematic eating behaviors, such as restrictive eating, binge eating, and associated compensatory behaviors (e.g., vomiting, laxative misuse, or excessive physical exercise). Often, but not always, these behaviors stem from body image concerns. Disordered eating may exist on a subclinical level or be a part of an overall clinical picture that fulfills formal diagnostic criteria for an eating disorder, such as anorexia nervosa, bulimia nervosa, binge eating disorder, or avoidant/restrictive food intake disorder (ARFID) (American Psychiatric Association 2013). Anorexia nervosa is characterized by restriction of energy intake leading to a significantly low body weight, intense fear of weight gain, and a disturbed experience of one’s own body weight or shape. Bulimia nervosa is characterized by recurrent episodes of binge eating, inappropriate compensatory behaviors to prevent weight gain, and excessive emphasis on weight or shape in self-evaluation. Binge eating disorder is characterized by recurrent episodes of binge eating without compensatory behaviors. ARFID is characterized by restrictive eating due to other factors than body image concerns, such as sensory selectivity, low appetite, or dysphagia. However, subclinical symptoms of disordered eating in the absence of a full eating disorder are significantly more common: during adolescence, around 24% of girls and 16% of boys report disordered eating (Hautala et al. 2008).

Anecdotal reports in blog posts and magazines have described how watching mukbang videos can trigger and reinforce disordered eating behaviors such as binge eating or purging behaviors, but that it may actually also help some individuals to restrain from binge eating or encourage overly restrictive eaters to grow an appetite and become more comfortable in eating in a social setting (Kim 2017; Quartz 2016). Hence, mukbang has been described as “a double-edged sword” in regard to disordered eating (Kim 2017): depending on the viewer context, it appears to be both potentially helpful and destructive. However, no formal scientific exploration of the mukbang phenomenon and its relation to disordered eating has been published.

In the current exploratory study, a netnographic qualitative content analytic approach was employed with the aim of understanding how viewers of online mukbang videos relate their audience experiences to symptoms of disordered eating, such as restrictive eating, binge eating, or purging behaviors. Specific questions that we wanted to explore were: Are mukbang videos perceived as influencing eating and/or purging behaviors in viewers? If so, in what ways are they described as triggering disordered eating behaviors? In what ways are they, on the contrary, described as potentially helpful for viewers in coping with and recovering from disordered eating?

## Methodology

Netnography was originally developed as a qualitative method for the investigation of consumer behaviors in Internet communities, with an emphasis on the researcher-as-participant (Kozinets 1998). Over the years, however, netnography has increasingly been used as a strictly observational approach in health care research, not least for studies on sensitive topics where it may be difficult to negotiate access or recruit informants for a more traditional qualitative design (Langer and Beckman 2005). A netnographic approach has been used in, for example, research on pro-anorexia communities (Dyke 2013) and online peer support in diabetes mellitus (Tenderich et al. 2019). The terms netnography, digital ethnography, and digital anthropology are sometimes used interchangeably. However, although the practical methodology may not differ very much, a key conceptual difference is that in a netnographic analysis, the online community under study is seen as a habitat in its own right, governed by sociocultural codes that are essentially separate from ‘offline’ life in many respects. In contrast, the digital ethnographer generally approaches an online community merely as an extension of or a complement to offline ethnographic studies. We do not wish to overstate these differences; even so, we believe that a number of unique features of mukbang are intimately connected to the online nature of the phenomenon and that a netnographic approach is therefore preferable.

In the present study, two modes of inquiry were employed. First, viewer comments of popular mukbang videos on YouTube were analyzed. YouTube (youtube.com) is a video-sharing platform that allows its users to upload, view, rate and comment on videos, most of which are user-generated. The wide variety of available content includes video blogs, live streams, music videos, short and documentary films, television clips, movie trailers, and educational videos. As of February 2019, YouTube is rated as the 2nd most visited Web site globally by Alexa Internet, Inc. (Alexa Internet 2019). Similar analyses of YouTube comments have previously been utilized in studies on topics such as non-suicidal self-injury (Lewis et al. 2012), weight stigma (Jeon et al. 2018), and pro-anorexia content (Oksanen et al. 2015).

On YouTube, the three most viewed videos from five popular mukbang hosts—i.e., a total of 15 videos (see Table 1)—were included in this analysis. These particular hosts were chosen mainly based on their popularity in terms of number of viewers, but also so as to reflect the geographical diversity of the mukbang phenomenon: as seen in Table 1, three of the hosts are based in South Korea, one in Japan, and one in the United States. Data on the basic characteristics of mukbang content in the 15 included videos are provided in Table 1. With the help of a free online tool (ytcomments.klostermann.ca) for downloading YouTube viewer comments in JavaScript Object Notation (.json) format, which is compatible with Microsoft Excel, the full comments section of these videos was retrieved on Feb 20, 2019 and then examined in close detail in search of viewer comments on the topics of disordered eating and body image. Here, an English-only language limit was applied. Naturally, this limits the transferability of the findings to other language contexts and we elaborate on the implications of this in the Discussion section. In total, over 174,000 YouTube comments were assessed. At this stage, any user comments relating to the research questions outlined above were included; thus, comments discussing symptoms of disordered eating (such as restrictive eating, binge eating, or purging behaviors), body image concerns, as well as the sociocultural role of mukbang were extracted for closer examination. In contrast, the large number of comments expressing amazement (e.g., “omfg yo”), admiration (e.g., “I love youuuu < 3”), or disgust (e.g., “Ewww ur grossing me out”) in a more general sense about the amounts of food eaten in the broadcasts were not included in the final qualitative analysis. As an illustration of the volume of comments excluded at this stage, around 200 variants of “I love cheese!”, several hundreds of “Hi!” and “Yummy!”, and well over 1000 versions of “R u gay???” were removed. These types of brief, nonsensical, or pejorative comments made up the bulk of the data.

**Table 1:** Characteristics of the assessed mukbang videos

Host	Country of origin	URL	Date posted	Length	Content	Number of views^a^	Number of comments^a^
Banzz	South Korea	youtu.be/pWkA6Wa-R0s	Dec 24, 2016	52 min	Host cooks and eats ten packages of ramen with canned meat, kimchi, and a bowl of rice	11,685,025	18,922
		youtu.be/N1YfXo7bWsY	May 4, 2017	20 min	Host partakes in a jjajangmyeon (noodles in blackbean sauce) challenge at a restaurant and then orders and eats another noodle dish with meat and seafood	11,656,464	11,756
		youtu.be/i73wZnkoNqo	Jun 22, 2014	28 min	Host cooks and eats five packages of ramen, around 20 dumplings, and an apple	9,659,126	8375
MBRO	South Korea	youtu.be/kehVg0eSYU8	Mar 9, 2016	81 min	Host eats around 20 pieces of roast chicken with large amount of rice	6,352,934	7850
		youtu.be/KPU_AJt9ktw	Apr 24, 2016	87 min	Host eats eight different dishes, including two plates of sushi, roast chicken, lobster, various noodle dishes, and a strawberry cake	5,693,652	6592
		youtu.be/qQxdZt-BGNU	Apr 27, 2017	87 min	Host eats around 40 pieces of fried chicken	4,952,336	3727
Shukii	South Korea	youtu.be/LF9N0Wxc7V4	Jun 3, 2015	31 min	Host eats three portions of tteokbokki (spicy rice cake in sauce), tuna mayo rice, cheese sticks, and fried fish cake	8,156,477	8394
		youtu.be/xJs-dWKJyFA	Nov 9, 2018	40 min	Host eats large pan of tteokbokki (spicy rice cake in sauce), several boiled eggs, five cheese sticks, and rice	4,160,608	8582
		youtu.be/G9v0tOHlgts	Jun 5, 2015	34 min	Host eats around 30 pieces of fried chicken with sauce	3,147,530	2134
Nikocado Avocado	United States	youtu.be/vytrCSYXi5w	Jun 1, 2017	32 min	Host cooks and eats four packages of ramen with butter and melted cheese and a slice of pizza	6,252,688	20,049
		youtu.be/I6f713Jr7qw	Aug 29, 2018	31 min	Host cooks and eats four packages of ramen with tteokbokki (spicy rice cake) and melted cheese	5,889,250	13,216
		youtu.be/VN-joeGLg1w	Jul 7, 2018	33 min	Host cooks and eats three packages of ramen and a large pan of tteokbokki (spicy rice cake)	5,874,311	14,967
Yuka Kinoshita	Japan	youtu.be/G68_hkc29po	Apr 2, 2017	10 min	Host prepares and eats six packages of ramen with a bowl of cheese and egg sauce	15,950,475	16,573
		youtu.be/SOpYWjAM91M	Feb 13, 2016	7 min	Host prepares and eats six packages of ramen with melted cheese and eggs	15,745,251	19,774
		youtu.be/T3XUfw95Kfg	Jul 28, 2016	4 min	Host prepares and eats large bowl of rice with 20 eggs and sauce	12,848,892	13,539

^a^Retrieved on Feb 20, 2019

Second, online posts on the topic of mukbang and disordered eating on Reddit were analyzed. Reddit (reddit.com) is a user-generated news and discussion Web site consisting of free-access topical or demographical discussion communities known as ‘subreddits’. As of February 2019, Reddit claims to have over 330 million monthly active users and is rated as the 17th most visited Web site globally by Alexa Internet, Inc., with 53.4% of its visitors originating in the United States making it the most popular English-language discussion community (Alexa Internet 2019). As a complement to the YouTube data, Reddit was chosen as an additional data source specifically because of its discussion forum format, which hypothetically encourages somewhat longer, more self-disclosing, and reciprocal comments. Reddit content has previously been analyzed in studies on various topics, such as suicidology (Aladağ et al. 2018), cannabis use (Sowles et al. 2017), pro-eating disorders communities (Sowles et al. 2018), and gout (Derksen et al. 2017).

For this study, fifteen relevant subreddits that contained discussions about mukbang were identified, utilizing the Reddit search function: r/1200isplenty, r/AnorexiaNervosa, r/BingeEatingDisorder, r/bulimia, r/confession, r/EDAnonymous, r/fasting, r/fatlogic, r/fatpeoplestories, r/food, r/fuckeatingdisorders, r/gainit, r/korea, r/loseit, and r/nutrition. In total, these subreddits had more than 19 million subscribers when data were retrieved on Feb 20, 2019. Data on user experiences of watching mukbang were collected by identifying specific posts or threads related to this subtopic with the help of the Reddit search function. Similar inclusion and exclusion criteria as in the analysis of YouTube comments were used. No time limits were applied, i.e., all comments available up to the date of data retrieval were included in the analysis.

It should be noted that both YouTube and Reddit allow users to respond to other users’ previous comments. In the data retrieved from Reddit, we could easily follow such exchanges. In the data retrieved from YouTube, however, this was not possible due to the fact that the.json data format did not allow for a hierarchical display of comments and subcomments. Even so, the context of the comments often made it possible to distinguish when a user responded to another user’s comment, as seen in the interactions described in the Results section.

The first pre-analysis phase of the assessment—i.e., identifying relevant YouTube videos and subreddits, retrieving comment data, and scanning these data for irrelevant or redundant information—was performed by the first author alone, due to the sheer number of comments and the largely mechanical nature of the work. In total, 1316 user comments (986 YouTube user comments and 330 Reddit user comments) were included in the final qualitative content analysis. The collected data were then analyzed using the qualitative analysis software NVivo 11. Qualitative content analysis is usually the preferred method of data analysis in netnographic studies (Langer and Beckman 2005); here, a directed approach to qualitative content analysis (Hsieh and Shannon 2005) was applied, guided by the research questions outlined above. Thus, data were primarily analyzed in a ‘top-down’ manner whereby any data relevant to our research questions were coded. When appropriate, a single comment expressing multiple or ambivalent statements could receive more than one coding label. In a second step, all relevant data were categorized in an iterative ‘bottom-up’ approach by the first author. Next, the second author independently coded all data in accordance with the preliminary coding scheme, in order to validate the categories. This step leads to some minor changes to the coding scheme, resulting in two overarching themes and 11 categories. An interrater reliability above 90% was achieved and any interrater disagreement was resolved by consensus.

The study was preregistered on the Open Science Framework (osf.io/4w32d). Since the study was of a strictly observational nature utilizing publically available online data, no ethical vetting was necessary according to Swedish law. In order to ensure full anonymity, no online user names (anonymous or otherwise) were collected, analyzed, or presented. For a more detailed discussion on research ethics in netnographic studies, see Langer and Beckman (2005).

## ‘Outsider’ and ‘Insider’ Perspectives

Two overarching themes, each consisting of several categories, were identified among the user comments. The first theme, which we have called *the viewer perspective*, consisted of 1022 comments that are made from an ‘outsider’ perspective (see Table 2). Here, users comment and discuss aspects of mukbang without describing any personal involvement. In contrast, in the second theme, which we have called *the participant perspective,* users take an ‘insider’ perspective in describing their own experiences of affect and behavior in response to watching mukbang videos (see Table 3). This second theme consisted of 269 comments, notably fewer than the first theme. Since all data were anonymized prior to analysis, we were not able to conclude whether individual users made multiple comments that fitted both of these overarching themes.

**Table 2:** Examples of complete user comments (i.e., comments have not been shortened or otherwise edited) from a viewer perspective

Category/subcategory	Examples of user comments
Viewer perspective	
1. Envy and amazement	Bruh she eat 8 cup rice 20 eggs and gains nothing I eat a peace of fruit and gain 457,867 zillion pounds
	How, just how the hell is this girl not fat???!!!! I simply breath and i gain five pounds!!! TF
	She eating like dinosaur but her body is very lithe …
2. Body shaming	He looks really good back then… i dont know what drives him to be as skinny as hes now. But really hes disturbingly skinny
	He have a double chin haha
3. Supportive	I would enjoy watching you eat a normal healthy meal. I love your personality and that has nothing to do with eating crazy hot, excessive calories, or anything that puts your health at risk
	Omg wtf is wrong with people nowdays…the ones that support that he has to change into a healthier diet, at least suport your opinion kindly with statements,not with not-funny-at-all insulting and rude coments about his diet or his appearence(tryna be savage or sth)…JEEZE think about what your saying and the way you say it. dont just hurt people
4. Explanations	
4a. Intensive physical exercise	He bikes over 200 miles a week and lifts 6 times a week
	Im not completely sure but ive heard that he works out more then half of his day and thats probably why hes so slim
	Actually he exercises 'bout 8 h per day to keep himself in shape. He said it on the live broadcast
4b. Restriction and purging behaviors	This sounds cynical but I wouldn't be surprised if most of them purged after eating. 10 k calories is a ton, especially if you are an influencer and need a perfect body. Heaps of mukbangers chew + spit/purge too
	I'm sorry but there is NO WAY she's not bulimic
	I'm annoyed at how skinny he is. How can he plow through more than a month's worth of food and not be obese? Unless he eats nothing but celery for the rest of the month… yeah that's probably it
	She never outright admits to having an eating disorder, in fact in one video she sits down and tries to differentiate the difference between what she does and eating disorders, stating that she’s “in control” and not “binging.” But she eats almost 10 k + calories each cheat day. That is a binge. She used to have them somewhat frequently, and she would always stay 120 lb. That’s because on the days she’s not filming herself stuffing her face, she HAS to be restricting or purging
4c. 'Medical mystery'	Her stomach is a mini Bermuda triangle
	Actually … she has a health condition where her stomach expands 6X, so in order for her body to absorb the nutrients she needs, she has to eat this much
	This guy has the most fastest metabolism system ever, i'm so jealous. i bet he never gets fat because all of the meat he eats, n shit goes to his height and probably feet
4d. Ethnicity	Because he is Asian lol… Some Asian including my family no matter how much we eat we hardly gain any weighs
	Nah man a ton of koreans are naturally skinny it's just how they are built it's just how you see skinny girls that don't gain what no matter how much they eat in America
5. Developing into a trend	Before the hype, it started out as videos of people (usually a woman) eating a meal in front of the camera so that lonely solo people (usually men) who come home to eat alone can feel like they are enjoying a meal with someone. Then Korean media got a hold of how popular these videos were and started making programs related to finding, cooking, and eating good food. And Korean celebrities also started making these videos of themselves eating. At no point was any of these videos and tv programs made for binge eating. At some point, Mukbang became an international thing (i blame K-pop) and then it transformed into some weird binge eating concept. And now here we are…
	This is why we can't have nice things. It started off so sweet and innocent to help lonely people…
	I follow some of these mukbangers, and damn, they are just a heart attack waiting to happen. It's always the same mantra, "You guys have been BEGGING me to film this (insert fast food) mukbang so I'm doing it for you guys!" No one is forcing you to gorge on 5,000 cal binges everyday. Don't act like what you're doing is somehow okay because your viewers ask for it. You are risking your health and wellbeing, and all the attention is just perpetuating the problem. I hope they see the light. Further, it appears that uploading these daily videos has become some people's main income stream, which is dangerous. Now you have to eat yourself to death to support yourself. Granted, these videos can be entertaining and it can feel like you're eating with friends. But maybe upload once a week, not every damn day!

**Table 3:** Examples of complete user comments (i.e., comments have not been shortened or otherwise edited) from a participant perspective

Category	Examples of user comments
Participant perspective	
1. Limits eating	I love watching mukbangs when I’m restricting. It brings me a satisfaction that they’re inhaling the calories and I’m not but also extreme jealousy and my mouth literally waters when I watch them sometimes. The high cal mukbangs are the best lol
	He eats I loose weight
	I'm eating through you lol
	to curb my appetite and stop myself from digging into a tub of ice cream… I genuinely feel sorry for them, but it also motivates me… a visualization of how I don't want to end up
	Makes me feel sick, but mainly when they're noisy about it. But it's enough to get rid of the craving I had. Lol, kind of like the house cleaning/hoarding shows make me immediately start cleaning my own home, even if it's not that dirty. Gives me that motivation
	It honestly might be kind of strange but watching them eat it is kind of cathartic and calms the urge to eat myself. It does make me hungry but for some reason it subdues the actual intention of going out and getting binge food
	I haven't eaten a real meal in 3 days so this is what I “eat”
2. Increases eating	Mukbangs made me binge more food in larger amounts it normalized it for me so I stopped watching them literally block the channels that come up in my recommended feed
	Funny that you mention this, after two years in recovery, watching mukbangs triggered me to relapse
	Great video, i'm trying to eat snacks from every country on Earth as a way to overcome my eating disorder, i've been underweight my entire life and am using youtube as a fun way to document my journey
	Your videos are so good for boosting appetite, helpful for me as I can' t eat when I should
3. Ambivalence	Mukbangs are hit and miss for me. Some days it inspires me to eat more, some days it makes me feel like restricting
	I think it does two things for me: 1. I get a vicarious satisfaction out of it (like, they're eating, so I don't have to), and 2. It more or less normalizes eating for me– I have a lot of weird shame around eating, like many people with eating disorders, and seeing other people eat without serious concern is vaguely validating. Idk. I still can't really fathom how people put up videos like that, though– just having people watch me eat normally is difficult, let alone binging haha
	I always feel kinda bad when I watch these videos. Maybe hypocritical is the right feeling. But at the same time it really does help me because it reminds me of what I could be.I feel sad for the people in those videos, too. Because I know now that they're struggling with an addiction
	It comforts my cravings while simultaneously makes me feel terrible about myself
	Lol do you have any recommendations to overweight mukbangers? i only see skinny korean girls doing this stuff and it makes me feel like shit lmao
4. Reduces loneliness	I totally get what you mean about the familiarity and closeness feeling, though. Mukbangs can definitely make you feel less lonely if it's done by someone whose personality you enjoy
	Feels like I'm having dinner with someone.:)
	I heard one person say that they watched people eat online because they used to eat family meals but his kids had grown up and his wife had left him. I thought that was a sweet reason to watch others eat
5. Reduces guilt about own eating	This is a relief, honestly! Youtube started recommending these videos a couple days ago, and maybe I'm trying (subconsciously I guess?) to normalize eating like a "normal" person. Sometimes I wonder how many calories are they eating or how do they burn that off, but most of them just carry on with their lives and that kinda brush off all the previous thoughts. Thanks!
	I'd do this exact same thing while I was recovering from anorexia! I'd watch "10,000 cal challenges" and other videos like this. They were my guilty pleasure while I was recovering. I think I watched them because I wasn't comfortable with eating that much food, so I'd just watch other people do it. I thought I was so weird for doing this, and I'd feel kinda ashamed afterwards. But it would help satisfy an urge ig, and these videos kinda helped normalize eating for me
	I watch these videos to feel better about the amount of food I eat
6. Obsessive and self-destructive	Meeeeeee omg I keep scrolling through it and watching mukbangs. Something I’ve never done before fasting
	When I discovered mukbang! The first channel I got into, I watched straight through an entire night literally laying there under the covers. It was pretty much like a binge, just…by proxy? I found it giving me this satisfaction without the suffering (aside from my eyeballs burning). I was into it for a while before I left to travel, where the habit followed but I at least had broken away from some detrimental factors like being home and bored etc. with a kitchen and privacy. At that point, mukbang sort of reminded me of darker times, so I phased it out

### The Viewer Perspective

Around 400 user comments describe *envy and amazement* over how mukbang hosts can eat such large amounts of food without apparent weight gain (approximately 400 comments; see Table 2, category 1). Many also express that if they would try the same, they would immediately gain weight in an undesirable way. A gender pattern can be observed in that most of these comments are directed at the two female hosts included in the assessment; however, because of the limited number of mukbang hosts included in the analysis, we did not attempt to perform any formal statistical analyses regarding quantitative comment patterns.

There are also a *body shaming* comments (approximately 200 comments; see Table 2, category 2). A few of these comments are made on the grounds of mukbang hosts being perceived as “too skinny”; however, a large majority of the body shaming comments are directed at the one host in our assessment who viewers mainly perceived as overweight and thus consisted in fat shaming. Still, there are a number of examples of users expressing a *supportive* view, calling out other comments as pejorative (approximately 20 comments; see Table 2, category 3). These supportive comments also often encourage mukbang hosts not to feel obligated to challenge themselves or try to impress viewers with excessive amounts of food, but to instead post videos of themselves eating more balanced meals for the sake of their own health.

Many users engage in more or less realistic *explanations* about how mukbang hosts are able to repeatedly ingest large amounts of food and seemingly not gain weight (approximately 270 comments). Here, several subcategories can be identified. *Intensive physical exercise* is mentioned as an explanation in a large number of comments (see Table 2, category 4a), although this topic is a subject of debate among viewers. Often, viewers point to the fact that some mukbang hosts themselves explicitly claim to spend several hours a day working out in order to be able to eat large amounts without gaining weight. However, some viewers question this assumption on the grounds that the hosts do not necessarily look very muscular or athletic. Instead, *restricting and purging behaviors* before and/or after the recording of mukbang videos are brought up as a more realistic explanation, albeit one that viewers assume that mukbang hosts would be more reluctant to admit (see Table 2, category 4b).

Yet another popular explanation among viewers is that mukbang hosts do not gain weight due to some type of ‘*medical mystery*’ (see Table 2, category 4c). Here, some users speculate about somewhat plausible explanations such as hypermetabolism or food passing undigested through the gastrointestinal system. Others, however, engage in highly unrealistic hypothesizing about unnamed rare medical conditions in which a person’s stomach is said to expand to the extreme to allow for the ingestion of excessive amounts of food (the numbers six or even 66 times that of a normal-sized ventricle is frequently mentioned, apparently inspired by a pseudo-medical television piece aired on a Japanese eating show). Another recurrent version of the medical explanation is that of East Asian *ethnicity* being associated with fast metabolism and a disposition toward a small body frame size, although this explanation is often dismissed as highly anecdotal by other viewers (see Table 2, category 4d).

Another viewer perspective category is that of how the mukbang phenomenon has successively been *degenerating into a trend* (approximately 30 comments; see Table 2, category 5). Here, commentators frequently explain the idea of mukbang having originated as a live substitute for the immensely important Korean family dinner, targeted at the increasing number of individuals in single-person households working long hours or being shut in their homes gaming or gambling online: “These people need to eat too! But they've been raised to feel absolutely uncomfortable eating by themselves, especially alone at home. So who steps in to fill this most unlikely of markets? Mukbang broadcasters of course!” A recurrent trope is that this benevolent online social eating community has subsequently been appropriated by sensationalist media and metamorphosed into a grotesque ‘voyeurist’ trend disconnected from its original social context. Whether or not this is an accurate depiction is beyond the scope of this article, but it provides a useful background for the participant perspectives described below.

### The Participant Perspective

Many users describe how watching mukbang makes them *limit their own eating* (approximately 150 comments; see Table 3, category 1). Individuals who appear to be restricting their eating in an unhealthy way recurrently attest to “eating vicariously” through the mukbang host, i.e., watching mukbang makes them feel that they themselves do not need to eat. Some users engaging in restrictive eating explain this in terms of being repelled by the large amounts of food ingested in the mukbang videos, thereby losing their appetite. A more common experiential description, however, is that of eating ‘by proxy’ and actually enjoying watching the mukbang host, all the while feeling proud about oneself abstaining from eating. The same overall pattern is found in comments made by users who appear to have a history of binge eating: they sometimes comment on how watching mukbang helps them stave off a binge by finding the amounts of food ingested in the videos repellent, but more often they too describe a more pleasant experience of calm in seeing someone else eat “for” them.

Others describe how watching mukbang *increases their own eating* (approximately 75 comments; see Table 3, category 2). Here, a majority of users appear to have a history of binge eating and attest to how watching mukbang videos may trigger them to relapse into loss-of-control eating. In contrast, a substantial minority of comments in this category appears to be made in a context of struggling with low appetite and/or selective eating and finding mukbang helpful as an inspiration in increasing food intake. Furthermore, many comments express *ambivalence* from a participant perspective toward the mukbang phenomenon (approximately 65 comments; see Table 3, category 3). For example, users mention that their response to watching mukbang varies, so that it sometimes leads to increased eating and at other times enforces restriction. Others describe how mukbang evokes several types of affects and behavioral impulses at once, e.g., being inspired to eat less in the moment while simultaneously reducing shame around eating in general, or that they enjoy the eating part of the mukbang concept but feel distressed about not being as thin as the mukbang host.

A number of comments relate to the idea of mukbang as being primarily a social phenomenon discussed above. Here, users describe how watching and participating in mukbang *reduces loneliness* and makes them feel like they are having dinner with someone (approximately 30 comments; see Table 3, category 4). Indeed, some individuals appear to develop a close (albeit one-way) online relationship to a certain mukbang host over time, praising their good-humored personality as a crucial factor in engaging in the mukbang eating community. These comments are sometimes made in conjunction with descriptions of how watching mukbang increases eating. Furthermore, users describe how mukbang *reduces guilt about their own eating* and that watching mukbang videos is helpful in normalizing eating (e.g., demonstrating that eating can be a joyful experience and that occasional overeating does not automatically need to evoke feelings of guilt; approximately 35 comments; see Table 3, category 5).

Finally, a number of comments mention that watching mukbang can become *obsessive and self-destructive* (approximately 65 comments; see Table 3, category 6). This is sometimes expressed primarily in the form of an excessive fascination with the peculiar conspicuousness of the mukbang concept, but also potentially as part of a pattern of disordered eating behaviors that reinforce the need to keep watching mukbang in order to maintain a restrictive diet. For some, it appears that mukbang can become a destructive way of reducing feelings of boredom.

## Discussion: Parasocial Interaction, Modernity, and Ambivalence

In conclusion, this study shows that users engage with online mukbang videos both as ‘outsider’ viewers and as ‘insider’ participants. In some instances, users discuss behaviors that are evidently part of a broader pattern of disordered eating. For example, they describe how they find mukbang useful in keeping a clearly restrictive diet or how watching mukbang may trigger a relapse into binge eating. However, there are also many examples of user comments that describe engaging in mukbang as a way of trying to increase food intake in a context of low appetite or selective eating, or as a way of actually preventing a binge eating episode.

The comments made from a participant perspective were often difficult to categorize in terms of ‘healthy’ or ‘unhealthy’. A comment about how watching mukbang makes the viewer eat more may point to mukbang as a helpful tool for an individual who is struggling with trying to increase the amounts and variety of food accepted and to gain weight. In contrast, the very same comment may be seen as a sign of the potential triggering and unhelpful nature of mukbang if made by an individual with a previous binge eating disorder who has now relapsed into loss-of-control eating. In some instances, the context in which a comment is made makes it possible to determine whether watching mukbang is helpful or unhelpful. More often, however, the context is opaque or ambiguous. We therefore chose to categorize comments made from a participant perspective in terms of whether watching mukbang results in the user limiting or increasing food intake, etc. As seen in Table 3, the examples of user comments vary in their description of contextual factors.

Notably, there is often a striking ambivalence in user comments on mukbang. The phenomenon is not necessarily seen as helpful *or* destructive, but instead simultaneously useful *and* hurtful. For some, it may evoke several types of affects and behavioral impulses at once, such as limiting food intake in the moment while simultaneously reducing shame around eating in general or experiencing a reduction in loneliness but also distress about the thin body ideal that is often promoted in the videos.

The present findings point to the explicitly social nature of mukbang. This sociality is probably best described in terms of so-called *parasocial interaction*. The concept of parasocial interaction was originally established in the 1950s to describe how viewers and audiences gradually develop a personal, albeit one-way, relationship to television celebrities, movie stars, and other public figures such as politicians (Horton and Wahl 1956). Subsequently, models of parasocial interaction have been applied in the analysis of a wide variety of mass media phenomena, from children’s identification with fictional television characters (Hoffner 1996) to charismatic relationships with televangelists (Diekema 1991). With the emergence of social media in the last decades, there has been a renewed interest in the concept. Indeed, a study commissioned by Google has shown that a majority of young people identify even closer with YouTube creators and video blog hosts than with traditional celebrities (O’Neil-Hart and Blumenstein 2016). Parasocial interaction develops over time as repeated ‘meetings’ with a performer make viewers experience increasing feelings of friendship and intimacy, much in the same way as they know and understand their flesh-and-blood friends (Ballantine and Martin 2005). Thus, viewing episodes may become an important part of the daily social life of the viewer. Not least, since online social media platforms often allow people to respond to celebrities’ messages, the relationship may come to be experienced as even more ‘real’ or intimate, even though it is (with very rare exceptions) non-reciprocal (Kim and Song 2016). This is probably even further exacerbated by the tendency of many online celebrities to self-disclose and to share everyday life experiences (which may, of course, include food and eating). In the case of mukbang, the non-reciprocity between host and audience may actually be less evident, given that viewers may sometimes donate money in virtual currency in real-time, whereupon they are usually named and thanked directly by the hosts.

There has been some debate about whether or not parasocial interaction should be viewed as a dysfunctional behavior—e.g., as a substitute for non-virtual interpersonal relationships. Some evidence suggests that parasocial interaction may help broaden the scope of an individual’s interpersonal relationships, rather than compensate for a lack of closeness (Ballantine and Martin 2005). The findings from the present study indicate that mukbang viewers may indeed develop parasocial relationships with mukbang hosts. Moreover, it seems that they often, if not always, experience these relationships as helpful in reducing loneliness or alleviating feelings of guilt. In the case of body image concerns and thin-ideal internalization, upward social comparison—such as the typical parasocial relation between fan and celebrity—has been shown to be a mediating factor (Fitzsimmons-Craft et al. 2014). However, research findings on parasocial interaction and body image are equivocal. One study showed that young women with a desire to be and look like a popular female television character do display more body image concerns. On the other hand, the actual level of parasocial interaction with that character is not associated with body shame or body surveillance (Greenwood 2009). Likewise, another study showed that men who are exposed to images of muscular superheroes tend to feel bad about their own bodies. However, those men that have a strong parasocial relation to said superhero (e.g., as a result of having spent a childhood immersed in comic books) do not display the same tendency and may actually feel better about themselves upon exposure (Young et al. 2013).

Another crucial aspect of mukbang is, undoubtedly, the massive amounts of food consumed. Here, the vital role of excess and exaggeration in the construction of a notion of ‘modern life’ has often been noted. For example, the concept of *conspicuous consumption*, introduced by sociologist Thorstein Veblen in the 1920s to explain the emergence of a tendency to uphold social status by display of consumption patterns and leisure time (Veblen 2009), could potentially be evoked to shed light on the mukbang performance. The gendered aspects of modernity, excess, and ‘the grotesque’ have also been emphasized (Russo 1994). Notably, many twentieth century theorists have tended to describe these phenomena in more or less dystopian terms: as commodity fetishism within a ‘society of spectacle’ (Debord 1992), as virtuality and hyperreality devoid of meaning (Baudrillard 1983), or as unavoidable aspects of a new media ecology (McLuhan 1964). A straightforward illustration of this pessimistic and cynical tendency is the 1973 French-Italian movie *La Grande Bouffe*, directed by Marco Ferreri. In this satire on consumerism and bourgeoisie decadence, a group of middle-aged male friends retreats to a villa over the weekend with the purpose of eating themselves to death—anticipating, perhaps, the excesses of twenty-first century mukbang.

A notable exception to this dystopian view of overconsumption and overeating is literary theorist Mikhail Bakhtin’s work on the medieval world of François Rabelais. Here, Bakhtin shows how the satirical yet joyous and affirmative depictions of carnivalesque banquets in Rabelais’ writing “differ sharply from the images of private eating or private gluttony and drunkenness in early bourgeois literature” (Bakhtin 1984, p. 302). A direct observation is that the mukbang performances included in the present study, weirdness and grotesquerie notwithstanding, have nothing overtly cynical about them. In all 15 assessed mukbang videos, the tone is friendly, intimate, and youthful. As described above, this parasocial quality may not be truly reciprocal and the mukbang phenomenon as such could certainly be critiqued from an anti-consumerist point of view. Even so, the actual performance of mukbang eating is uniformly depicted as the host taking on and joyously conquering the enormous amounts of food rather than the food-as-commodity triumphing over the consumer.

A more accurate comparison, then, may be so-called *food porn*; i.e., carefully staged images of appealing dishes that are consumed visually on blogs or in glossy magazines rather than viscerally at the dining table. The consumption of food porn has been described as “ingestion without incorporation” (Lavis 2015, p. 201)—an elusive confusion of virtual and actual eating. This very quality may contribute to the fascination with food porn, cook books, and recipes that individuals with anorexia nervosa often display as a way of constantly engaging with food without actually eating it (in the everyday meaning of the word). Just as food porn shared on blogs or social media may come to function primarily as a social relationship between people rather than as simply a collection of alluring images (Lavis 2015), it should be clear from the findings of the present study that the mukbang phenomenon is more than just a display of someone eating large amounts of food. Indeed, for some viewers with disordered eating, mukbang appears to fill the very same (para)social function of ‘ingestion without incorporation’ as do food porn.

This ambiguous stance toward mukbang, seen in many of the analyzed comments, mirrors a broader ambivalent tendency in disordered eating and body dissatisfaction. It has often been noted that ambiguity is at the very core of an eating disorder such as anorexia nervosa: “[Patients] experienced pleasure and disgust, were empowered and disempowered, felt safe yet constantly threatened, were both pure and dirty, and when sickest felt at their best. Anorexia was a constant process of becoming and unbecoming, of having a life by moving toward death.” (Warin 2010, p. 4) Not least, feeling in control while also being controlled by the illness is a very common theme in patient interviews: “It takes control of you, but it can also feel very safe. It’s a very confusing illness, because at the moment it’s probably got a lot of control over me, in certain ways, and I just want to get away from it, I’m just sick and tired and I’m exhausted, but then it kind of protects you as well, I think, from coping with other things.” (Tan et al. 2003, pp. 632–633) This ambivalence is often depicted as two opposing forces involved in an inner struggle for command over the recovery process (see for example Gorse et al. 2013). It may also involve equivocal feelings about the ‘food as medicine’ paradigm and psychiatric medication (Lester 2014), based on ideas that restrictive eating, for instance, is actually a morally virtuous forms of self-care rather than an alarming symptom (Musolino et al. 2015). However, others have described an ambiguity of a more profound nature in eating disorder narratives, involving breaks with self-continuity and linear recovery trajectories in favor of circular timelines, uncertainty in terms of selves and self-mastery, and irony (Shohet 2007, 2017).

## From ‘Sweet and Innocent’ to Carnivalesque

As seen in Table 1, the content of the analyzed mukbang videos in terms of the food eaten was surprisingly homogenous. A substantial majority of the broadcasts involved eating large amount of ramen noodles, regardless of geographical origin (South Korea, Japan, or the United States). Although this is certainly not always the case, there appears to be a fascination among viewers with the supposedly Asian features of mukbang. In our analysis, we found a recurrent lamentation about the “sweet and innocent” Korean origin of mukbang (i.e., social eating online targeted at single households) that has subsequently transformed into “an international thing” and, consequently, “some weird binge eating concept” (see Table 2, category 5). Even though further exploration of the successive development of the mukbang phenomenon is beyond the scope of this article, it can be noted that the prevalence of disordered eating and body dissatisfaction has been shown to be relatively high in a South Korean context (Jackson, Keel, Lee 2006; Jung and Forbes 2007; Pike and Dunne 2015).

From a ‘technical’ point of view, mukbang can be described as a pre-planned binge eating episode staged and performed for a virtual audience (sometimes also with a monetizing purpose). Indeed, the explicit language of ‘bingeing’ is often used in viewer comments and would appear to be a vital factor in the alluring appeal of the phenomenon. Here, comparisons can be made with ‘competitive eating’ or ‘speed eating’ shows, where participants compete in eating as much as possible in front of an audience and a series of judges during a specified, usually brief, period of time. Interestingly, such eating contests have been described as combining the spectacle of the medieval carnival with modern ideas of consumerism and abundance (Johnson 2011). For example, the annual Nathan’s Hot Dog Eating Contest held in Coney Island, New York City, has been aired on live television to millions of viewers since the early 2000s. Likewise, it has been recognized that popular descriptions of eating disorders often display an ambivalent and uncanny fascination with the ‘carnivalesque’ image of thinness and illness (Warin 2004). Such connotations may deserve more attention from the research community in the context of online video broadcasting.

To the best of our knowledge, this is the first published study of the mukbang phenomenon from a perspective of disordered eating behaviors and body image. A very large number of YouTube comments and Reddit posts were analyzed during the assessment phase and a high interrater reliability in terms of the identified thematic categories was achieved. Still, the exploratory nature of the study and the small-scale scope of the included video material (i.e., 15 broadcasts from a total of five mukbang hosts) limit the transferability of the findings. For example, although the overall impression during coding was that saturation was reached in the data, we cannot exclude the possibility that including other mukbang hosts and/or videos in the analysis would yield a slightly different result. As described above, any hypothetical gender or geographical patterns could not be formally explored due to the limited scope of the raw data. Furthermore, whereas the analysis was limited to comments and posts in English, there were also many YouTube viewer comments in Korean, Japanese, Chinese, and other languages. Due to the sheer volume of comments and the explorative nature of the study, it was not possible to have the non-English comments translated. Naturally, this limits the transferability of the findings to other language contexts, although it was obvious that many of the English comments included in the analysis were made by non-native English speakers from all over the world. Future research on the topic of mukbang should build on this explorative study and extend the analysis to include a broader scope of mukbang hosts, video material, and languages.

Another challenge for future research is to somehow negotiate access to the mukbang community in order to conduct formal interviews with viewers/participants as well as with mukbang hosts about their experiences within the scene. The netnographic approach used here is advantageous in research on sensitive topics where it may be difficult to negotiate access or recruit informants for a more traditional qualitative study. However, the approach naturally does not allow for an in-depth exploration with follow-up questions, informant clarifications, etc.

Furthermore, due to the inherently subjective nature of online user comments, we cannot know with any certainty that users that describe disordered eating actually suffer from a clinically relevant eating disorder. We do not wish to exaggerate any clinical implications of our findings; nevertheless, clinicians working with patients with eating disorders should be aware of the mukbang phenomenon as a potentially influencing factor.

## Conclusion

In sum, this qualitative study adopting a netnographic approach shows that online users engage with the mukbang scene both as ‘outsider’ viewers and as ‘insider’ participants. The participant perspective includes descriptions about how watching mukbang may make some users limit or increase their own eating in a highly context-dependent manner. Not least, there is often a striking ambivalence in how users are influenced by and make sense of mukbang. For some, mukbang appears to be a constructive tool in increasing food intake, preventing binge eating, or reducing loneliness. For others, it is clearly a destructive force that may motivate restrictive eating or trigger a relapse into loss-of-control eating. Perhaps most notably, the mukbang phenomenon is not necessarily seen as either helpful *or* destructive, but instead as simultaneously useful *and* hurtful.
